# Macular disease research in the United Kingdom 2011–2014: a bibliometric analysis of outputs, performance and coverage

**DOI:** 10.1186/s13104-015-1825-1

**Published:** 2015-12-30

**Authors:** Pamela Royle, Norman Waugh

**Affiliations:** Division of Health Sciences, Warwick Medical School, University of Warwick, Coventry, CV4 7AL UK

**Keywords:** Macular disease, Age related macular degeneration, Bibliometrics, Citations, Research priorities

## Abstract

**Background:**

Bibliometric indicators, based on measuring patterns of publications and citations, are widely used by universities and research funders to assess research performance. Our aims were to: (1) perform a bibliometric analysis of UK macular disease research publications from 2011 to 2014 and compare this with the other countries producing major output in the area, and (2) compare the pattern of UK macular disease publication with the priorities for age-related macular degeneration (AMD) developed by the Sight Loss and Vision Priority Setting Partnership (SLV-PSP).

**Methods:**

We used the Scopus database to retrieve macular disease articles published from 2011 to 2014. Citations to articles from 2011 to 2013 and journal impact factors (JIFs) for 2014 articles were obtained. Articles with UK authors were allocated to the 10 SLV-PSP priorities for age-related macular degeneration (AMD), where possible.

**Results:**

The UK, USA, and Germany and China were the top four producers of macular disease research from 2011 to 2013. All except China had a higher proportion of citations than articles. There were 421 articles with UK authors published from 2011 to 2014, of which 49 % had international collaborators. The UK produced 9.7 % of the world’s output of macular disease articles from 2011 to 2013, but received 14.2 % of the world’s share of citations. UK authors’ share of the world’s top 10 % of cited publications from 2011 to 2013 was 16.2 %. In 2014, 13.2 % of UK articles were in journals in the top 10 % when ranked by Journal Impact Factors (JIFs), while the overall UK article share for that year was 9.9 %. UK articles did not show a strong correlation between citations and JIFs. The SLV-PSP published a set of 10 priorities for research into age-related macular degeneration in October 2103. Only 8 % of the UK’s 2011–2014 publications matched the SLV-PSP top priority (treatment to stop dry AMD progressing) and 34 % did not match any of the SLV-PSP priorities, mainly because the priorities did not include invasive treatment of wet AMD.

**Conclusions:**

The UK is performing well in macular research, based on bibliometric indicators. The distribution of past research topics does not match the priorities set by the SLV-PSP.

## Background

Macular disease is a major cause of morbidity in the UK. Age-related macular degeneration (AMD) is the commonest cause of visual loss in people aged over 65. Owen and colleagues have estimated the UK prevalence and incidence of late AMD, in the population aged 50 and over, at 2.4 % in 2007–2009 [1]. The prevalence increased to 4.8 % in those aged 65 years or more. They estimated that in 2020 the number of AMD cases will be 679,000.

Hereditary retinal dystrophies, including Stargardt disease, were the commonest single cause of visual loss in working age adults in 2009–2010, accounting for 20.2 % of blindness certifications in people of working age in England and Wales [2].

There have been recent advances in treatment of wet (neovascular) AMD but not in dry AMD or Stargardts and more research is needed. The Sight Loss and Vision Priority Setting Partnership (SLV-PSP) developed a “top 10” list of research priorities for AMD in 2013 [3].

### Bibliometric measures of research performance

Bibliometric indicators are based on measuring patterns of authorship, publication and citations, and are widely used to evaluate the research performance of researchers (both individually and as a group) and of institutions. Such indicators form a key part of the global ranking and league tables which are important to Universities. For example, one such ranking, the *Times Higher Education* (THE) World University Rankings 2015–16, used 13 performance indicators, grouped into five areas [4]. Research influence, measured by citations, is weighted at 30 percent of the overall score. Another indicator was research productivity, accounting for 6 % of the overall score. This is based on a count of the number of papers per person (scaled for institutional size and normalised for subject) which were indexed in peer-reviewed journals in Elsevier’s Scopus database. Another indicator, accounting for 2.5 % of the total score, is international collaboration; that is, the proportion of a university’s total research journal publications that have at least one international co-author.

Rather than looking at citations to the total output of a department, institution or country, some evaluations judge the quality of research based on the number of highly-cited papers produced. This is based on the assumption that the most highly-cited articles are likely to have made the greatest contribution within their field, or to be about important innovations. Highly cited is variously defined as being in the top 20 % [5], or top 10 % [6], or top 1 % [7] of citations.

### Journal Impact Factors (JIFs)

The JIF of a journal for a particular year is the average number of times articles from the journal have been cited in the past 2 years [8]. The JIF is often used to indicate the prestige of the journal, and publication in journals with a high JIF is often regarded as an indication of the quality of the paper itself. Consequently, there is considerable pressure on academics to publish in high JIF journals [9, 10].

However, some researchers and authors have called for a stop to the practice of JIFs being used as a surrogate measure of the quality of individual research articles when assessing an individual scientist’s contributions, or in hiring, promotion, or funding decisions, and for research to be assessed on its own merits [11].

### Research needs and prioritisation of macular research

The James Lind Alliance (JLA) Priority Setting Partnerships (PSPs) are part of the National Institute for Health Research (NIHR) [12]. The aim of the PSPs is to identify and prioritise unanswered scientific questions that they agree are most important, and to help ensure that funders of health research are aware of the priorities of patients and clinicians.

In 2012, the Sight Loss and Vision Priority Setting Partnership (SLV-PSP) surveyed patients, carers and health care professionals to identify and prioritise unanswered questions in the prevention, diagnosis and treatment of sight loss and eye conditions [13]. This process resulted in the identification of the top questions for research for each of the 12 eye disease/condition categories, including the top 10 priorities for AMD.

## Aims

The aims of this report are: (1) to use bibliometric indicators to review the UK’s research performance in macular disease conditions from 2011 to 2014 and to compare it with other countries producing major output in the area, and (2) to compare the UK’s publications with the SLV-PSP’s top 10 priorities for AMD.

## Methods

### Database searching

We compiled a database of journal articles on age-related macular disease (AMD) and Stargardt disease. The latter was chosen to represent the retinal dystrophies because it is the commonest (there are over 100 inherited retinal diseases and we were not resourced to include them all in this study) [14]. For the sake of brevity we will refer to AMD and Stargardt collectively as macular disease, though as will be seen, the great majority of research articles relates to AMD.

### Database searching for macular disease articles from the UK, USA, China and Germany

We searched Elsevier’s Scopus database on February 18, 2015 using the search terms “stargardt* or age related macular degeneration” in the Article Title, Keyword or Abstract fields and limited it to publication years from 2011 to 2014. Only document types indexed as ‘articles’ or ‘reviews’ were downloaded. Editorials, letters, comments and notes were excluded. The search was not restricted to human research, but could include, for example, stem cell research in animals.

All documents, without any country limit, were downloaded. The search was then repeated and refined using the Country field limits (in turn) to: (1) United Kingdom (2) United States (3) Germany and (4) China. This limited the searches to those articles with at least one author with an address in these countries. The searches were downloaded separately into Excel. The fields downloaded included; Authors, Title, Year, Title of Journal, Volume, Cited by (number of citations), Affiliations of authors to institutions (including institution of the author for correspondence), Abstract and keywords and Document Type (article or review).

### Database searching for macular disease articles from the UK

An additional search, for UK authored articles only, of the Thomson Reuters Web of Science™ *(WoS)* database was done to check whether any relevant records were missed by the Scopus search (possibly due to differences in indexing practices and journal coverage between the two databases). The *WoS* search strategy was: TOPIC: (age related macular degeneration OR stargardt*) *AND* ADDRESS: (north* ireland or scotland or england or wales) and limited to Document Types: (article or review) and Timespan: 2011–2014.

The results from the UK authored articles for Scopus and WoS, were combined and duplicates removed.

### Classification of UK macular disease research into AMD SLV-PSP priorities

All 421 articles in the UK dataset were allocated where possible to the ten SLV-PSP priorities by one author (NW), initially based on title and abstract. In case of doubt the full text of articles were obtained and checked. Articles corresponding to the SLV-PSP priority 1 were sub-divided into reviews or primary research.

### Percentile ranking of citations and JIFs

We obtained the top 10 % of world citations separately for 2011, 2012 and 2013 by downloading citations to macular disease articles and ranking the articles on the basis of citations in Excel and then selecting those articles in the top 10 % of journals.

To obtain the articles in the 10 % of the top ranked journals for 2014, we downloaded all the 2014 macular disease articles, obtained the JIFs of the journals they were published in, ranked them by their JIFs, and selected those articles in the 90th percentile. We used the 2013 Journal Impact Factors, obtained from the Journal Citation Reports^®^, published in June 2014.

### Statistical analyses

We used non-parametric statistical tests to analyse data, due to the non-normal distribution of the dependent variables, citations and JIFs. The Wilcoxon Mann–Whitney Test was used to analyse the difference in citations between two independent groups, the Kruskal–Wallis test was used to analyse the differences in citations between three or more groups. Spearman’s correlation coefficient was used to compute the correlations. Stata 14.0 (Stata Corporation, College Station, TX, USA) was used for the analyses.

## Results

### Bibliometric analysis of UK macular disease publications from 2011 to 2014

We retrieved bibliographic records of 431 articles in Scopus when limiting the search to the United Kingdom. Thirty-two articles were deleted, as macular disease was not the prime focus of the article, and this left 399 articles. The WoS search identified another 22 articles, giving a total of 421 in the final UK data set: 81 % were indexed as articles and 19 % as reviews.

We analysed citations only to 2011, 2012 and 2013 articles, because many 2014 articles would not have had time to accumulate sufficient citations at the time we did the searches. The citations were analysed separately for each of the 3 years (to account for the fact that each year had a different citation window).

The numbers of citations to the UK articles from 2011 to 2013 are shown in Table 1.

**Table 1:** Citations to UK publications for 2011, 2012 and 2013

Year	Number of articles	Number of citations					Percentage uncited (%)
		Median	IQR	Mean	Range	Mean per year	
2011	96	9.0	4–17.5	15.4	0–128	3.85	6.3
2012	96	7.5	3–16.5	14.4	0–196	4.80	8.3
2013	119	4.0	2–8	7.6	0–96	3.80	13.4

*Mean* citations for all years, *Mean per year* overall mean divided by the number of years in which citations were possible

The highly skewed nature of the citation distribution is evident from the data. A small number of articles received a large number of citations and the vast majority received relatively low numbers. To adjust for the fact that the older articles had more time to accumulate citations, the mean number of citations per article per year was calculated by dividing the mean citations per article by the number of years between publication and collection of the citation data. The mean number of citations per year was 3.9 % for 2011 articles, and 4.8 and 3.8 per year for 2012 and 2103 respectively. The percentage of articles that received no citations (6.3, 8.3 and 13.4 % for 2011, 2012 and 2013 respectively) was low (when considering the citation windows for each year), and will be likely to decrease over time.

### Journals in which UK authors publish

The 421 UK macular disease articles were published in 141 different journals.

Table 2 shows the top 4 journals, which published 27.8 % of the articles. The first and third ranked journals (*British Journal of Ophthalmology* and *Eye* respectively) are from the UK and the other two (*Investigative Ophthalmology & Visual Science* and *Ophthalmology*) are based in the USA.

**Table 2:** Top 4 journals in which UK MD articles were published

Journal	Number	Percentage	JIF
British Journal of Ophthalmology	33	7.8	2.809
Investigative Ophthalmology and Visual Science	31	7.4	3.661
Eye	29	6.9	1.897
Ophthalmology	24	5.7	6.170

### International collaboration

Forty-nine percent of articles were from international collaborations, with the number of countries ranging from 2 to 12. The most frequent collaborating country was the USA (22.3 %) followed by Germany (11.6 %), and Australia (7.1 %). Seventy-two percent of articles had the corresponding author based in the UK. The highest proportion of non-UK corresponding authors were the USA (10.9 %) and Germany (4.3 %).

### Correlation between JIFs and citations

We examined the Spearman’s correlation between citations and JIFs for 2011, 2012 and 2013. In those years, 92 % (285 of 311) articles were in journals with a JIF. The results in Table 3 show that the strength of the relationship between citations and JIFs is highly statistically significant for all years, but varies over the 3 years. There is a weak correlation for 2012 (rho = 0.3526), and moderate correlations for 2011 (rho = 0.4635), and 2013 (rho = 0.6036).

**Table 3:** Correlation between citations and JIFs

	Number of articles	Correlation coefficient	P value
2011	85	0.4635	<0.0001
2012	91	0.3526	0.0006
2013	109	0.6036	<0.0001

### Comparing UK macular disease output and citations with other countries

The Scopus searches for macular disease articles worldwide from 2011 to 2013 retrieved 3262 articles. The four countries with the highest output (USA, UK, China and Germany) produced 64 % of the world’s output of macular disease research that we identified from 2011 to 2013. The world share of citations for these four countries was calculated and compared to their share of total citations, shown in. It can be seen that the USA, UK and Germany have higher percentage of the world share of citations than their share of the world output of articles, and are performing above the world average in terms of citations. China has a lower proportion of citations than articles, and on this measure is performing less well (Table 4).

**Table 4:** Citations versus output from 2011 to 2013 in macular disease research for USA, UK, Germany and China

	USA	UK	China	Germany
Proportion of world output	36.8 %	9.7 %	9.3 %	8.4 %
Proportion of world citations	54.9 %	14.2 %	6.8 %	10.8 %
Ratio of citations to output	1.49	1.46	0.73	1.29

The ratio in the last row is the ratio of proportions of outputs and citations

### Proportion of UK Authors publications in highly cited publications

We looked at the proportion of UK authored papers included in the top 10 % of the world’s most cited macular disease articles. The total numbers of macular disease articles in the Scopus database were 1007 in 2011, 1121 in 2012, and 1139 in 2013 respectively. The numbers of citations needed for an article to be in the top 10 % of world citations in macular disease for these years were 28, 20 and 11 respectively.

Table 5 shows the proportion of highly cited publications with a UK author for 2011, 2012 and 2013, the UK’s overall article share for each year, and the ratio of highly cited articles to article share. It shows that in 2011, 2012 and 2013, UK authors’ share of the highly cited publications were 1.37, 1.88 and 1.78 times respectively greater than their share of world articles in macular disease.

**Table 5:** Proportion of UK authors of highly cited publications compared to article share

	2011	2012	2013
UK authors on highly cited articles (top 10 % by citations)	13.7 %	16.5 %	18.3 %
UK overall article share	10.0 %	8.8 %	10.3 %
Ratio of UK authors on highly cited articles to overall article share	1.37	1.88	1.78

The highly cited articles with UK authors comprised 76 % (41/54) primary research (mainly concerning treatment and pathophysiology) and 24 % review articles.

### Articles in the top 10 % of JIFs in 2014 from UK authors

The 1135 worldwide macular disease articles published in 2014 journals were ranked by JIF. Eighty percent (906 of 1135) were in journals with a JIF, and 229 were in journals with no JIF. If we assume that in all articles with no JIF, that JIF = 0, and rank them on their percentiles based on JIF we find that the JIF needed to be in the top 10 % of journals 5.939. The mean JIF was 2.802 and median was 2.512.

We found that in 2014, 13.2 % of articles in journals ranked in the top 10 % of JIFs in macular disease had a UK author. UK authors’ world share of macular disease articles for 2014 was 9.9 %.

### Matching UK macular research to the sight loss and vision priority setting partnerships (SLV-PSP)

We matched the set of 421 UK publications on macular disease from 2011 to 2104 against the SLV-PSP priorities for AMD. The results are in Table 6 (note that the numbers added up to 423 as there were 2 articles that belonged to both the categories ranked 5 and 6). Thirty-four percent of the publications did not fit any of the SLV-PSP priorities, as they did not include invasive treatment of wet AMD in their list. Only 8 % of the publications were on the top priority of the SLV-PSP list, which was treatment to stop dry AMD progressing and/or developing into wet AMD. Forty-four percent of publications addressing the top priority were primary research and 66 % were reviews.

**Table 6:** Comparison of published research and SLV-PSP priorities

Rank	Priority	Number	Percentage
1	Can a treatment to stop dry AMD progressing and/or developing into the wet form be devised?	34	8.0
2	What is the cause of AMD?	96	22.7
3	How can AMD be prevented?	2	0.5
4	Are there ways of restoring sight loss for people with AMD?	1	0.2
5	Can the development of AMD be predicted?	19	4.5
6	What is the most effective way to detect and monitor the progression of early AMD?	54	12.8
7	What factors influence the progression of AMD?	1	0.2
8	Can a non-invasive therapy be developed for wet AMD?	11	2.6
9	Can dietary factors, nutritional supplements, complementary therapies or lifestyle changes prevent or slow the progression of AMD?	23	5.4
10	What are the best enablement strategies for people with AMD?	11	2.6
	Articles that do not match any of the JLA priorities. Most were concerned with wet AMD	144	34.0
	Uncertain	1	0.2
	Stargardt’s dystrophy—the SLV-PSP list was only for AMD	26	6.1

The second priority, ‘What is the cause of AMD?’, had 23 % of the publications, largely because of the amount of research into the genetics of AMD. This category was defined by the SLV-PSP as including whether the genetic factors responsible for the development or progression are known. Studies of genes comprised 45 articles, the biggest component of this group. It could be argued that genes are not the cause of AMD, but merely a factor resulting in susceptibility to it. The real causes remain to be determined. Similarly we included 19 studies on the role of complement under group 2, but this could be more a mechanism of disease triggered by the primary cause. It could be argued that the gene studies belong under group 5 (predictors) or group 7 (factors influencing progression).

If we take out the gene and complement studies from group 2, and take into account that some of the remaining articles are reviews, the body of literature on causes of AMD in the dataset begins to look rather sparse.

The third and fourth ranked PSP priorities, prevention of AMD and ways of restoring sight loss, matched only two and one publications respectively.

## Discussion

We performed a bibliometric analysis of UK macular research from 2011 to 2014, and compared UK performance to the other leading countries in macular research, i.e. the USA, Germany and China. Also, we compared the 421 UK macular disease research publications published between 2011 and 2014 with the SLV-PSP’s top 10 priorities for AMD. The UK, USA, and Germany all performed above the world average in terms of citations compared to their article share, whereas China had a lower proportion of citations than articles. The mean citations per article per year for UK macular disease articles from 2011 to 2013 was 4.15. Forty-nine percent of articles were internationally collaborative. The UK was shown to be performing well, producing 9.7 % of the world’s output of macular disease articles from 2011 to 2013, but receiving 14.2 % of the world’s share citations. Also, 16.2 % of the top 10 % of macular disease publications, ranked by citations, had a UK author. In 2014, 13.2 % of articles in journals ranked in the top 10 % of JIFs were from UK authors; the overall UK article share was 9.9 %.

When matching UK macular research published from 2011 to 2014 (but performed earlier) against the priorities chosen in 2013 by the Sight Loss and Vision Priority Setting Partnerships (SLV-PSP), we found a considerable difference. Only 8 % of the publications were on the top priority of the SLV-PSP list and 34 % of the publications did not fit any of the SLV-PSP priorities.

### Strengths of this study

Comprehensive searches of Scopus and the WoS were done to identify as much as possible of the world’s scholarly output on macular disease, and all abstracts were checked by both authors. We set our bibliometric performance of UK macular research in a world context, by comparing it to the other three top producers of research in this area. We also included examination of the mostly highly cited publications in the field, which has been recognised as a robust approach to research assessment [7, 15].

We measured citations in early 2015 to articles published between 2011 and 2013, thereby giving a citation window ranging between 2 and 4 years. Although articles will still be accumulating citations, in most fields a paper is thought to reach its citation peak at 3 years and for citations to decrease quickly thereafter [6, 16]. Liu and colleagues [17] examined time to citations of articles in 28 ophthalmology journals. They reported that there were few citations in the first year after publication, but a rapid increase later, reaching a peak in year 3.

Finally, we compared UK macular research with SLV-PSP priorities to see how well matched the research needs that had been identified by consensus amongst patients, carers and clinicians.

### Limitations of this study

Our searches done using the terms ‘age-related macular disease or Stargardt*’ may have missed relevant research if neither term was used in title, keywords or abstract of the article. However this would not affect international comparisons. We did not look at other retinal dystrophies.

Although citations are a good indicator of research quality, they are not perfect. While there has been much debate about their meaning, they are still widely used in the assessment of academic performance of individuals and institutions [5, 7, 18, 19]. Some bibliometric studies exclude self-citations, but we included them in our analysis, as we took the view that this is a valid scientific practice (e.g. authors providing background and building on their previous work) [16].

### Bibliometric analysis of macular research

In this study of macular research, we found that from 2011 to 2013 the UK produced 9.7 % of the world’s output, but received on average 14.2 % of the world’s share citations. Overall for these years the mean number of citations per article was 4.15. This is compared to the aggregate impact factor (AIF) of 2.357 for journals in the Journal Citation Reports^®^ subject category of Ophthalmology for 2014 (the AIF is calculated in the same way as the JIF but at the subject level). This reflects well on UK macular research and shows that it is performing above average compared to the world-wide average for articles in ophthalmology journals.

In terms of the ratio of its share of world citations compared to output, for UK’s ratio of 1.46 compared favourably to the USA (1.49) and Germany (1.29). However, China’s ratio (0.73) was the lowest of the four countries. This concurs with findings from a recent study by Huang et al. who analysed articles published in 53 ophthalmic journals from 2000 to 2011, and found that despite the rapid increase in output, China ranked low in terms of citations to articles in ophthalmic journals [20].

### UK authors and highly cited publications

From 2011 to 2013, UK authors had an average of 16.2 % of their macular disease articles in the top 10 % of the world’s most cited publications in this area, with only 9.7 % of the share of articles. This over representation of UK authors (including those with international collaboration) in the world’s top cited publications reflects well on UK macular disease researchers.

Our findings are consistent with those found in a 2013 report by Elsevier on all UK research. The Elsevier report was commissioned by UK’s Department of Business Innovation and Skills (BIS) to examine how the UK research base compares internationally and what trends may affect the UK’s future standing as a world-leading research economy [7]. They found that while the UK produced 6.4 % of global articles in 2012, its share of global citations was 11.6. Also, the UK produced 15.9 % of the world’s most highly cited articles (defined as being in the top 1 %). It therefore concluded that the UK punches above its weight in terms of citations.

### UK authors and JIFs

In 2014, 13.2 % of articles in journals in the top 10 %, ranked by JIF, were from UK authors, compared to the overall UK article share of 9.9 %, showing that UK authors were over-represented in these journals s, in comparison with their article share. Despite objections to JIFs being used as a surrogate measure of the paper itself [21], and the fact many articles in journals with high JIFs are never cited [6, 22, 23], publishing in a journals with a high JIF is nevertheless considered prestigious, and very important for an academic’s reputation and career advancement [9].

### Uncited articles

Another indicator of performance is the proportion of papers that are never cited, which a report by Thomson-Reuters uses to identify publications with no or very little influence [24]. We found that on this measure UK macular research did well. Larivière et al. [25] estimated that the proportion of articles uncited in the medical field in the WoS database was 20 % after 2 years and 12 % after 5 years. In this study, we found that the percentage of UK articles uncited was 6.3 % for those with a citation window of just over 4 years, and 8.3 and 13.4 % for 3 and 2 year citation windows respectively. Therefore, UK macular research compares favourably on this indicator.

### International collaboration

Our study found an international collaboration rate of 49 % in UK macular research. The BIS report also found that UK researchers are highly collaborative, and in 2012 47.6 % of all UK articles involved international collaboration [7]. They noted that international research collaboration and researcher mobility were core to the maintenance and further development of the UK’s world-leading position as a research nation, but that increased collaboration and research leads to a lessening of differences amongst countries [7].

### Correlation between citations and JIFs

We found that the correlation between citations to the macular disease articles and the JIFs of the journals in which they appeared from 2011 to 2013 varied from low to moderate over the 3 years. This lack of a consistent and strong correlation is similar to other recent reports which have examined the correlation between JIFs and citations to articles [26–28]. Ramin et al. found that the number of citations correlated poorly with the impact factor of journals in the AMD field [28]. This provides further evidence against using JIFs as a proxy for citations to individual articles in the journal.

### Comparison of research published in 2011–2014 and the SLV-PSP’s research priorities

We found a considerable difference between the topics in the research published in 2011–2014 and the SLV-PSP priorities. It should of course be noted that the SLV-PSP report was published in October 2013, by which time the research documented in this report would have been done. The report of the SLV-PSP envisages that it will help funders of research into AMD to allocate research funding to the top priorities [3]. This assumes that researchers will also target those priorities, but the report suggests that they will be encouraged to do so and that future applicants for research grants should say which of the priorities their proposed research will address. The 2015 Vision 2020 funders meeting concluded that researchers should be made more aware of the SLV-PSP priorities, and that researchers should state which of the priorities their research would address [29].

Time will tell whether the prioritisation exercise affects allocation of research funds and the priorities of researchers. There may sometimes be a tension between patient priorities and the sorts of research that universities want done, but one would expect that research into the top SLV-PSP priorities would be attractive to universities. The balance of research will reflect the availability of treatments. In wet AMD there are several treatments, with research underway looking at how best to use them. In dry AMD, research is at an earlier stage.

A bibliometric study by Ramin and colleagues looked further back in time, at 3235 articles on AMD published between 1993 and 2013, identified via the Web of Science [28]. They found that most of the highly-cited papers were on genetics, treatment of wet AMD, and on the effects of diet and vitamins. They noted a recent increase in studies in biomarkers including genes.

## Conclusion

In terms of publications and citations as a measure of scientific quality, the UK has done well in macular disease research. The UK is one of the leading countries in macular research.

However the distribution of past research topics does not match the priorities set by the SLV-PSP in 2013, and future funding may encourage a different pattern of research.

## Abbreviations

AMD: age-related macular degeneration
BIS: Department of Business Innovation and Skills
JIF: Journal Impact Factor
SLV-PSP: Sight Loss and Vision Priority Setting Partnership
UK: United Kingdom
USA: United States of America

## References

[1] Owen CG, Jarrar Z, Wormald R, Cook DG, Fletcher AE, Rudnicka AR (2012). The estimated prevalence and incidence of late stage age related macular degeneration in the UK. Br J Ophthalmol.

[2] Liew G, Michaelides M, Bunce C (2014). A comparison of the causes of blindness certifications in England and Wales in working age adults (16–64 years), 1999–2000 with 2009–2010. BMJ Open..

[3] Sight Loss and Vision Priority Setting Partnership. Setting priorities for eye research—final report. 2013. http://www.sightlosspsp.org.uk/. Accessed 27 Oct 2015.

[4] Times Higher Education. The times higher education 100 under 50 rankings 2015. 2015. https://www.timeshighereducation.co.uk/world-university-rankings/2015/one-hundred-under-fifty-/. Accessed 27 Oct 2015.

[5] van Leeuwen T, Grant J, Chonaill SN. Bibliometric analysis of highly cited publications of health research in England, 2002–2006. RAND Europe working paper series 2011. http://www.rand.org/content/dam/rand/pubs/working_papers/2011/RAND_WR829.pdf. Accessed 27 Oct 2015.

[6] Ismail S, Nason E, Marjanovic S, Grant J. Bibliometrics as a tool for supporting prospective R&D decision-making in the health sciences: strengths, weaknesses and options for future development: RAND Technical Report (TR-685-DH). 2009. http://www.rand.org/pubs/technical_reports/TR685.html. Accessed 27 Oct 2015. 10.7249/rhq-01-04-11 PMC4945260 28083218

[7] Elsevier. International Comparative Performance of the UK Research Base—2013: a report prepared by Elsevier for the UK’s Department of Business, Innovation and Skills (BIS). 2013. https://www.gov.uk/government/uploads/system/uploads/attachment_data/file/263729/bis-13-1297-international-comparative-performance-of-the-UK-research-base-2013.pdf. Accessed 27 Oct 2015.

[8] Thomson Reuters. The Thomson Reuters impact factor. 2015. http://wokinfo.com/essays/impact-factor/. Accessed 27 Oct 2015.

[9] Frank M (2015). Impact factors-the bane of our existence. Physiologist..

[10] Werner R (2015). The focus on bibliometrics makes papers less useful. Nature.

[11] American Society for Cell Biology. San Francisco declaration on research assessment 2015. http://www.ascb.org/dora/. Accessed 27 Oct 2015.

[12] James Lind Alliance. James Lind alliance priority setting partnerships. 2015. http://www.jla.nihr.ac.uk/home. Accessed 27 Oct 2015.

[13] Rowe F, Wormald R, Cable R, Acton M, Bonstein K, Bowen M (2014). The Sight Loss and Vision Priority Setting Partnership (SLV-PSP): overview and results of the research prioritisation survey process. BMJ Open..

[14] Smith J, Ward D, Michaelides M, Moore AT, Simpson S (2015). New and emerging technologies for the treatment of inherited retinal diseases: a horizon scanning review. Eye (London, England)..

[15] RAND. Bibliometrics: Key findings from a report on the theory and practice of bibliometrics in health research. 2012. http://www.rand.org/content/dam/rand/pubs/research_briefs/2012/RAND_RB9684.pdf. Accessed 27 Oct 2015.

[16] Bornmann L, Benjamin L, Bowman BF, Bauer J, Marx W, Schier H, Cronin B, Sugimoto CR (2014). Bibliometric standards for evaluating research institutes in the natural sciences. Beyond bibliometrics: harnessing multidimensional indicators of scholarly impact.

[17] Liu XL, Gai SS, Zhang SL, Wang P (2015). An analysis of peer-reviewed scores and impact factors with different citation time windows: a case study of 28 Ophthalmologic Journals. PLoS One.

[18] Wouters P, Thelwall M, Kousha K, Waltman L, de Rijcke S, Rushforth A, et al. The metric tide: literature review (Supplementary Report I to the Independent Review of the Role of Metrics in Research Assessment and Management). 2015.

[19] Wilsdon J, Allen L, Belfiore E, Campbell P, Curry S, Hill S et al. The metric tide: report of the independent review of the role of metrics in research assessment and management. 2015. http://www.hefce.ac.uk/pubs/rereports/Year/2015/metrictide/Title,104463,en.html. Accessed 27 Oct 2015.

[20] Huang W, Wang W, Zhan J, Zhou M, Chen S, Zhang X (2013). Scientific publications in ophthalmic journals from China and other top-ranking countries: a 12-year review of the literature. BMC Ophthalmol..

[21] Alberts B (2013). Impact factor distortions. Science..

[22] Albarran P, Crespo JA, Ortuno I, Ruiz-Castillo J (2011). The skewness of science in 219 sub-fields and a number of aggregates. Scientometrics..

[23] Testa J. The Thomson Reuters Journal selection process. 2012. http://wokinfo.com/essays/journal-selection-process/. Accessed 27 Oct 2015.

[24] Thomson Reuters. Using bibliometrics: a guide to evaluating research performance with citation data. 2008. http://ip-science.thomsonreuters.com/m/pdfs/325133_thomson.pdf. Accessed 27 Oct 2015.

[25] Larivière V, Gingras Y, Archambault E (2009). Brief communication: the decline in the concentration of citations, 1900–2007. J Am Soc Inform Sci Technol.

[26] Finardi U (2013). Correlation between Journal Impact Factor and citation performance: an experimental study. J Informetr..

[27] Lozano GA, Larivière V, Gingras Y (2012). The weakening relationship between the impact factor and papers’ citations in the digital age. J Am Soc Inform Sci Technol.

[28] Ramin S, Soheilian M, Habibi G, Ghazavi R, Gharebaghi R, Heidary F (2015). Age-related macular degeneration: a scientometric analysis. Med Hypothesis Discov Innov Ophthalmol..

[29] VISION 2020 UK. VISION 2020 UK FundERS of Eye Research Summit 14 October 2015: Presentations and notes. http://www.vision2020uk.org.uk/vision-2020-uk-funders-of-eye-research-summit-2015-presentations-and-notes/. Accessed 27 Oct 2015.

